# Moral Judgment Among Chinese Urban and Rural Students: Exploring the Conflict Between the Personal and Moral Domains

**DOI:** 10.3390/bs15020187

**Published:** 2025-02-11

**Authors:** Fei Ye, Jie Chen, Hui Li, Huajie Liu

**Affiliations:** 1 Institute of Moral Education, Nanjing Normal University, Nanjing 210023, China; 02425@njnu.edu.cn; 2 School of Educational Sciences, Nanjing Normal University, Nanjing 210023, China; chenaca@163.com; 3 School of Education, Guangzhou University, Guangzhou 510006, China; 4 College of Teacher Education, South China Normal University, Guangzhou 510631, China; 20141057@m.scnu.edu.cn

**Keywords:** Chinese students, U-shaped development pattern, moral judgment, personal domain, moral domain

## Abstract

The U-shaped development pattern in social cognitive domain theory can explain adolescents’ “moral retrogression” in a certain period. This study employs quantitative methods to investigate moral judgments made by Chinese urban and rural junior high school students in situations involving conflicts between the personal and moral domains, thereby cross-culturally validating the proposed U-shaped pattern. The results show that this pattern exhibits some degree of cross-cultural applicability. Specifically, grades 8–9 represent a transitional phase in the moral development of Chinese students from both urban and rural areas. Students in the grade 9 tend to defend their personal domain when in conflict with the moral domain. However, the content and scope of the moral and personal domains may vary across cultures. When urban students encounter conflicts between the personal and moral domains, they tend to protect the personal domain. Furthermore, Chinese students have a strong sense of self-defense awareness, and when the target of their behavior is an immoral person in a specific context, it significantly increases their support for non-moral behaviors. This knowledge may be valuable for educators and policy-makers seeking to design moral educational programs aimed at promoting moral behavior among adolescents.

## 1. Introduction

Moral judgment is the process of using moral concepts or knowledge to evaluate actions as right or wrong, and it is the key for teenagers’ everyday moral decision-making. Previous studies have shown that adolescents in middle childhood (5–11 years old) exhibit more flexible and nuanced moral judgments (Jambon & Smetana, 2014). Research on experimental psychology and cognitive neuroscience has also demonstrated that there are substantial group disparities in children’s moral reasoning abilities between the childhood period (6–8 years old), pre-adolescent period (9–11 years old), early adolescent period (12–14 years old), and mid-adolescent period (15–17 years old). Moreover, in situations of moral conflict, adolescents not only chose the hedonistic alternative significantly more often than adults but also demonstrated a greater increase in activity in the frontal areas, the middle temporal gyrus, the thalamus, and the parahippocampal gyrus compared to adults when faced with everyday social conflict situations that required a decision between a social-oriented behavior and personal need (Sommer et al., 2014).

For this kind of phenomenon in adolescents’ moral development, especially the tendency of middle school students to make moral judgments based upon individual utilitarianism, previous studies have indicated that the U-shaped development pattern in social cognitive domain theory can explain adolescents’ “moral retrogression” that occurs within a specific age range (10–14 years) (Nucci, 2001; Nucci et al., 2018). However, this pattern has been verified only by empirical studies conducted in the United States (Jambon & Smetana, 2014; Dahl et al., 2018). Its applicability to the general development of adolescent morality across different cultures, particularly non-individualistic cultures, thus requires further empirical support.

Mainland China, as a prototypical collectivist country, is characterized by significant differences between urban and rural areas. Previous research on moral judgments of Chinese adolescents has revealed disparities between rural and urban samples (Yang & Wang, 2018). Therefore, the present study employs quantitative methods to explore moral judgments made by Chinese urban and rural junior high school students in the context of conflicts between the personal and moral domains with the goal of cross-culturally validating the U-shaped pattern. Investigating the moral development of Chinese adolescents based on the U-shaped developmental pattern is highly important with regard to both the advancement of this theoretical model and the examination of the moral development of adolescents in urban and rural contexts from a novel theoretical perspective.

## 2. Literature Review

The social cognitive domain theory divides social cognition into the moral, social, and personal domains. With respect to the moral domain, adolescents develop distinct patterns of moral thinking that are separate from considerations of prudence, self-interest, or social conventions beginning in early childhood (i.e., at approximately 4 years old). In the personal domain, judgments regarding behaviors do not implicate others’ interests and lack both formal and informal normative constraints, thereby granting individuals the right to make autonomous decisions, such as choosing friends, making decisions regarding entertainment options, or expressing their personal preferences (Nucci, 2001). Furthermore, studies (Nucci, 2001; Smetana, 2002) have demonstrated that adolescents’ personal domain develops through a process of growth. From late primary school to high school, adolescents broaden their definitions of the personal domain and gradually gain the freedom they need to self-govern. The domain theory posits that adolescents’ moral judgments at all ages result from the rational coordination of various situational considerations rather than being driven by mechanical or emotional factors (Turiel, 2008).

As such, moral judgments and decisions vary with the situational context. Moral decisions can involve interactions between moral and non-moral considerations and goals as well as interactions among distinct moral goals. The development of the ability to coordinate various considerations when making evaluations and decisions is key to a child’s development of moral judgments (Nucci & Turiel, 2009; Nucci et al., 2018). Therefore, when a situation involves only one moral goal and does not require coordination with other moral or non-moral goals, adolescents of different ages can make judgments that satisfy the moral goal in question. In situations that involve multiple considerations, adolescents’ moral judgments exhibit a U-shaped development pattern. In situations that require choices between moral and non-moral goals, primary and high school children tend to focus on moral goals, whereas nearly half of the middle school children sampled in previous research (who were approximately 14 years of age) prioritized their personal autonomy and individual rights in line with the notion of self-interest (Nucci & Turiel, 2009; Nucci et al., 2018).

The universality and cultural heterogeneity of moral judgment have always been the focus of international debate. Recent academic studies focus mainly on the similarities and differences of moral judgment under collectivist culture and individualist culture. Some studies have shown that moral concepts and judgments are generally the same across cultures: A study analyzing samples of adolescents (ages 12 to 20; N = 9112) from five contexts (North Macedonia, Mexico, Taiwan, the United Kingdom, and the United States) showed that the concepts of virtues among teenagers in different cultures have all developed, and their judgments on the concepts and behaviors of virtues are largely similar (Thoma et al., 2019). Some studies believe that the universalism framework may lead to the underestimation of the role of culture in moral reasoning, that the universalist framework may lead to an underestimation of the role of culture in moral reasoning, and that the processes that underlie moral cognition may not be a human universal in any simple sense because moral systems may play different roles in different cultures (Sachdeva et al., 2011). Empirical studies on Chinese adolescents’ moral judgments have indicated that these adolescents tend to adopt more traditional ethical perspectives in the moral domain than their Western counterparts. Chinese adolescents are more likely to exhibit altruistic tendencies and highlight the need to maintain in-group harmony, which are core values advocated in collectivist cultures, than American adolescents, who often express egocentric concerns (Zhang & Li, 2015). Studies comparing the sociomoral reasoning of children and adolescents aged 7, 9, 12, and 15 years old from two collectivistic cultures (Spain and Russia) in the 1990s have shown that self-interest tends to decrease with age, whereas altruism tends to increase (López-Pérez et al., 2015). Other studies have shown that there are certain cultural differences in specific moral content (Hu et al., 2020), corresponding to the predecessors’ research results demonstrating the universality of the staged development of moral judgment while specific moral characteristics are influenced by different cultures (Fang et al., 2003).

Concerning the cross-cultural applicability of the domain theory, numerous studies have demonstrated that individuals can distinguish among the moral, social, and personal domains from an early age (Nucci, 2001; Killen et al., 2002; Turiel, 2002). However, the scope and content of the individual domain can vary across cultures (Smetana, 2002), thus implying that adolescents from different cultures may make different judgments concerning whether a given issue can be independently decided by an individual (Zhang & Li, 2015; Hu, 2018; Hu et al., 2020). Only limited empirical research has investigated the cross-cultural applicability of the U-shaped moral development pattern posited by the domain theory.

Moreover, there are studies suggesting that culture plays a significant role in promoting and spreading moral judgments and behaviors, and differences in moral culture within a society may be as important as the differences across societies (Graham et al., 2016; Zhao & Selman, 2020). Within Chinese society, significant disparities have been reported between rural and urban students’ moral judgments. For example, urban students are more likely to endorse self-determination when they encounter conflicts with authorities or issues related to nurturance than rural students (Lahat et al., 2009). In terms of values, urban middle school students focus more on the “self”, whereas rural children often emphasize “relationships” with others (Yang & Wang, 2018). These findings suggest that urban students are more likely to prioritize individual interests and the realization of self-value than rural students. These empirical results are in line with the perspective expressed by some scholars that the moral values exhibited by individuals in rural areas are more honest and kind-hearted. However, some studies have also reported that urban adolescents obtain higher scores than rural adolescents in some moral dimensions (Yang & Wang, 2018). In particular, previous studies on the moral issues associated with rural left-behind children (i.e., children who remain at home while their parents work in other locations for an extended period) have revealed that these children tend to lack moral cognition and exhibit weakened moral quality, which leads them to engage in improper moral behaviors, exhibit character defects, and adopt distorted values (Chi, 2005; Zhu, 2007; Du, 2012). Additionally, a study that investigated left-behind children’s cognition across various domains via the domain theory revealed that they struggle to determine the scope of personal responsibility and individual autonomy and are more likely to accept rule denial in the personal domain than non-left-behind children (Liu & Yang, 2017).

To sum up, the U-shaped development pattern in social cognitive domain theory has been verified only by empirical studies conducted in the United States. Whether it can be used to explain the universal state of children’s moral development still requires the support of empirical research in non-American and non-individualistic cultures, especially paying attention to the differences among different groups within society. The focus of this study is the following two questions: What are moral judgments of urban and rural teenagers like in China, a non-individualistic culture? Does the U-shaped pattern of social domain theory still have universality among urban and rural adolescents in China? The answers to these questions enable us to have a clearer understanding of the characteristics and differences in moral judgment among Chinese teenagers, especially those in urban and rural areas. This knowledge may be valuable for educators and policy-makers seeking to design moral educational programs aimed at promoting moral behavior among adolescents, especially regarding some obvious selfish behaviors and other moral issues that emerge during the junior high school stage.

## 3. Materials and Methods

### 3.1. Sample

The sample investigated in this study was conveniently drawn from urban and rural regions in China: Nanjing in Jiangsu Province, which is a well-known historical and cultural city in eastern China, and Jishui County in Ji’an City, Jiangxi Province, which is located in central China. Jishui County was chosen because it includes a large proportion of left-behind children, which represents a typical background for rural children in China (left-behind children in rural areas account for more than one-third of the total population). Cluster sampling was used to select six classes from grades 7, 8, and 9 (i.e., two classes from each grade) in a school in Nanjing that employed a nine-year system. The same method was used to select six classes of rural students in a township school in Jishui that also employed a nine-year system. The total sample was 605; the number of urban students was 262, accounting for 43.31% of the total, while the number of male students was 316, accounting for 54.39% of the total. These students’ ages ranged from 11 to 17 years (M = 13.57, SD = 1.13). Based on the number of students in grades and classes in Chinese schools, this study was conducted with 95% confidence and an allowable error of 5–6%, with the number of urban and rural classes and grades kept as close to each other as possible. The effective questionnaire rate in the survey was nearly 100%, so no post-weighted adjustment was made.

### 3.2. Data

This study assessed moral judgments made by Chinese junior high school students via a research tool proposed by Nucci et al. (2018). In light of the primary goal of this study, which is to validate the U-shaped pattern proposed by a previous study conducted within a Chinese cultural context, the scenarios used in this research were altered only in terms of the names of the actors to align with Chinese cultural expressions. Meanwhile, this study transformed Nucci’s semi-structured interview guideline into a questionnaire to facilitate the collection of a large quantity of data.

This research tool featured 27 (3 × 3 × 3) situations: three actions (direct harm, indirect stealing, and helping) were presented within three contexts (unconflicted, self-conflicted, and other conflicted) in light of three possible recipient relationships (general other, vulnerable other, and antagonist other). Actions that involve causing direct harm to others are viewed as violating moral obligations, whereas preventing harm is generally regarded as a universal moral obligation. In this study, the situation concerning indirect stealing involved the possibility of acquiring someone else’s property and required participants to decide whether to return money to someone who had unknowingly dropped it. Helping others in distress is generally viewed as a “prosocial” action, as opposed to actions involving theft or violence. Each of the three actions that this research focused on was depicted in three distinct contexts. In the first context, the actor had no competing goals that could influence the decision-making process (e.g., hitting someone for no reason). In the second context, declining to engage in the action in question resulted in conflict with the actor’s goals (e.g., hitting someone in self-defense). In the third context, a third person’s goals conflicted with the actor’s decision not to act (e.g., hitting someone to protect another child). The students who participated in this research considered each of these situations in the following order: general other, vulnerable other, and antagonist other.

Regardless of the theoretical foundations or results of empirical research, these 27 situations can, to some extent, reflect middle school students’ moral judgments, thus ensuring content validity. Moreover, previous research has reported significant situational differences and age differences in moral reasoning and judgments pertaining to these 27 situations among students, thus indicating the discriminant validity of this research tool. Verifying the results of previous empirical research conducted within a Chinese cultural context is one objective of this study. The theoretical foundation of the distinction between the moral and personal domains in the context of children’s social cognition has been supported by some cross-cultural studies, particularly within a Chinese context (Liu & Yang, 2017). Furthermore, the 27 situations included in the questionnaire are also very common in Chinese junior middle school students’ daily lives. Therefore, the questionnaire used in this study is not fundamentally different from the research tool used by Nucci.

### 3.3. Analysis

Statistical analysis was conducted via Stata/SE14.0 software. For each event, an ordered multiple logistic regression model was used to evaluate the effects of the situation, context, relationship, grade, and region on questions that focused on making judgments concerning right and wrong with regard to an action. Binomial logistic regression was used to evaluate the effects of the same variables on questions that focused on making judgments concerning the right to engage in the action. The model analysis aimed to obtain the estimated value of the coefficient “b” and to test the significance of the coefficient with the ultimate aim of determining the significance and direction (positive or negative) of the effects of each variable.

Four models were constructed with regard to the data pertaining to each issue. In Models (1) and (2), gender was included as a control variable, and dependent and situational variables were incorporated into the logistic model with the goal of investigating the significance of situational effects (two models were divided into non-interactive terms and interactive terms). Models (3) and (4) incorporated variables pertaining to the geographic region and grade into the previous models with the aim of investigating the significance of their main effects and interaction effects (i.e., two models were developed, one of which featured no interaction term and the other of which featured an interaction term).

## 4. Results

### 4.1. Descriptive Statistics

**Figure 1:**
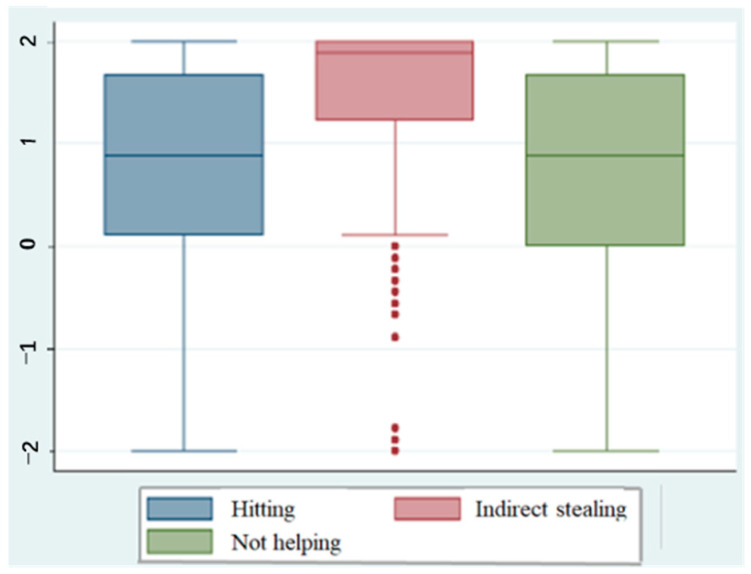
Distribution of judgments concerning rightness or wrongness.

Table A1 displays the percentage of participants who judged the protagonist’s actions to be wrong in different situations; the numbers are divided between urban and rural students across the three grades included in this research. The data revealed a significant disparity between the judgments associated with indirect stealing and those pertaining to the other two issues; regardless of the situation, a high proportion of participants judged the action in question to be wrong (average 87%). In contrast, the proportions pertaining to the issues of hitting and not helping were heavily influenced by situational variables. Descriptive statistics were used to calculate the average scores regarding the judgments concerning the rightness or wrongness of each issue, which are illustrated in Figure 1. This figure reveals that the scores associated with judging the action of indirect stealing were significantly different from those associated with judging the actions of hitting and not helping. In terms of judgments towards hitting and indirect stealing, the proportion of rural students in grades 7 and 8 judging the action to be wrong was generally lower than that of urban students. In particular, the proportion of judgments in situations in which the recipient was an antagonist differed. The proportion of students judging the acts to be wrong in grade 9 was also different between urban and rural areas, which was manifested in the judgments of every issue and every situation.

**Figure 2:**
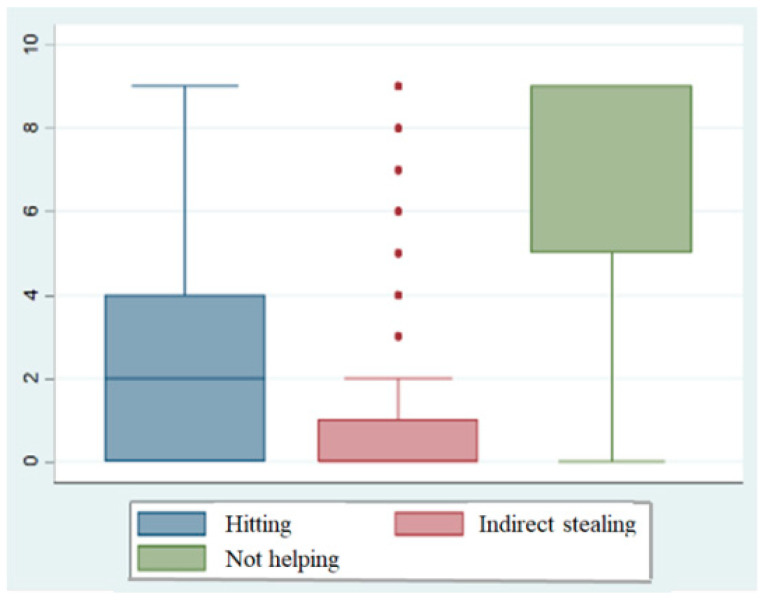
Distribution of judgments concerning rights.

The descriptive statistics concerning the percentage of participants who judged that the protagonist did not have the right to engage in the actions in question across the different situations are presented in Table A2 for both urban and rural students across the three grades included in this research. The data indicated that students’ judgments concerning the right to engage in a particular action differed among the three issues presented in this context. The average score for students’ judgments concerning the rightness of each action was calculated by reference to descriptive statistics, thus generating Figure 2. The data, as illustrated in Figure 2, revealed that the action in question had a significant effect on students’ judgments concerning the rightness of that action. The magnitude and significance of the difference between urban and rural students and between different grades also varied due to different issues and situational characteristics. Therefore, the significance of the corresponding difference was tested in the subsequent analysis of each action judgment.

### 4.2. An Ordered Multiple Logistic Regression Model

#### 4.2.1. Moral Judgments Concerning the Issue of Hitting

An ordered multiple logistic regression model was developed on the basis of the scores associated with each question. The variables and their interaction terms were successively incorporated into the model, and four models were developed (see Table 1).

**Figure 3:**
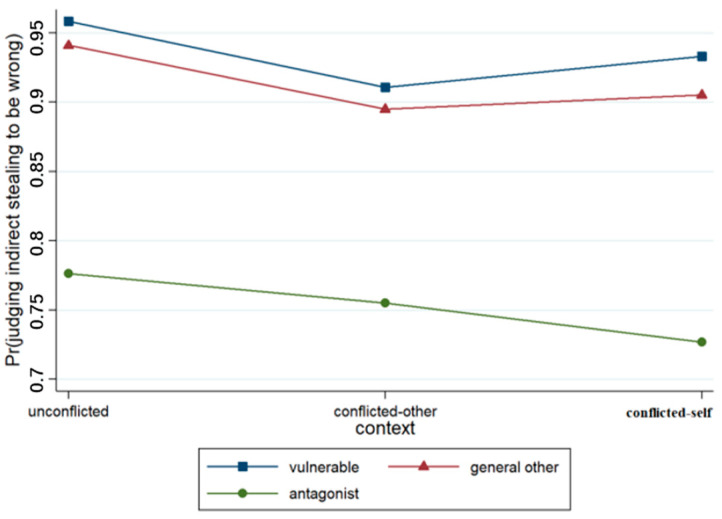
Interaction effect of context × relationship.

In Model 1, only the control variable of gender and two situational variables were included. The result indicated that the context (*p* < 0.001) and relationships (*p* < 0.001) had significant effects on the judgments made by junior high school students. The likelihood of students rejecting harmful acts decreased with the emergence of conflict as well as a change in the recipient of the acts from a vulnerable other to an antagonist other. The results regarding Model 2 indicated that the context × relationship approach was highly significant (*p* < 0.001). As shown in Figure 3, in the unconflicted situations, there was no significant difference between junior high school students’ judgments towards hitting the vulnerable and the general other. However, within the other conflicted contexts, there was no significant difference in judgments about hitting the general other or the antagonist. The majority of students judged hitting the vulnerable as wrong, but only about half of the students judged hitting the general and antagonist as wrong; within the self-conflicted contexts, the effect of relationship on moral judgments was further expanded.

**Figure 4:**
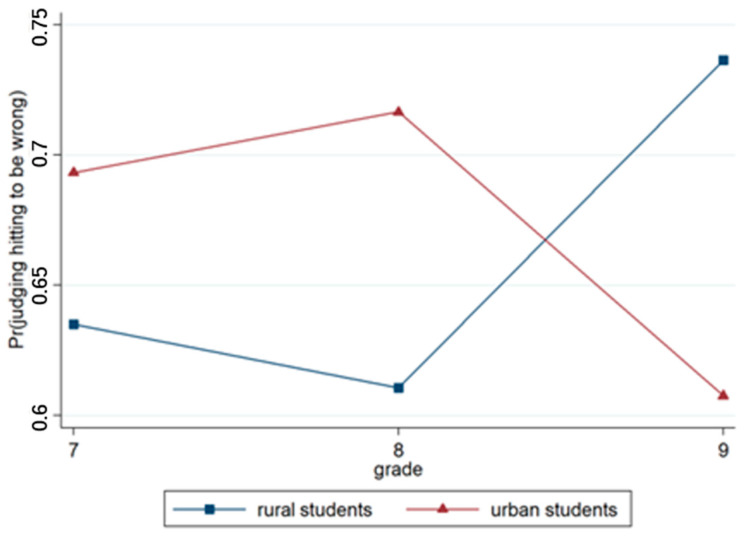
Interaction effect of region × grade.

Model 3 incorporated region and grade variables based on Model 2. Model 4 included the region × grade interaction terms. The output results regarding Model 3 indicated that the effects of region and grade were not significant. The results concerning Model 4 indicated that the region × grade approach was also highly significant (*p* < 0.001). As illustrated in detail in Figure 4, the difference between judgments of urban students and rural students was significant for different grades.

**Figure 5:**
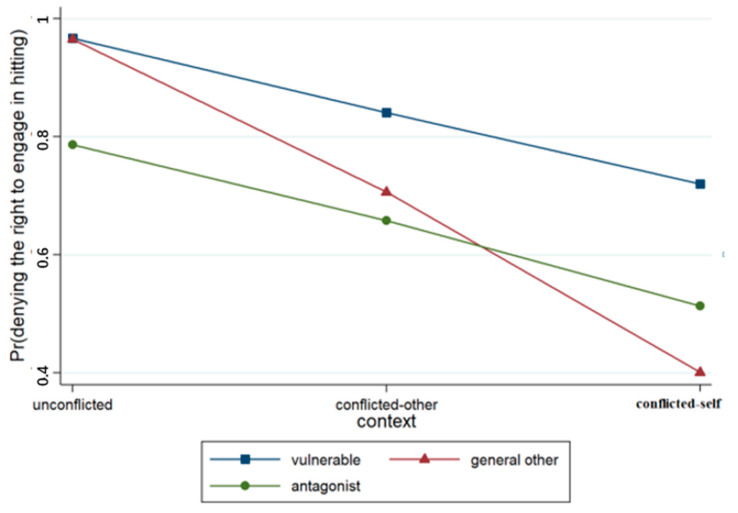
Interaction effect of context × relationship.

A binary logistic regression model was developed with regard to the judgment concerning the right to engage in hitting; the variables and their interaction terms were successively incorporated into the model, and four models were obtained (see Table 2). Model 1 included only the control variable of gender and two situational variables. The results revealed that context (*p* < 0.001) and relationship (*p* < 0.001) significantly impacted the students’ judgments and that the likelihood of affirming the right to engage in hitting increased with the emergence of conflict as well as a change in the recipient of acts from a vulnerable other to an antagonist. Model 2 included the context × relationship interaction term, and the results revealed that the context × relationship term approached significance (*p* < 0.001), as illustrated in detail in Figure 5.

**Figure 6:**
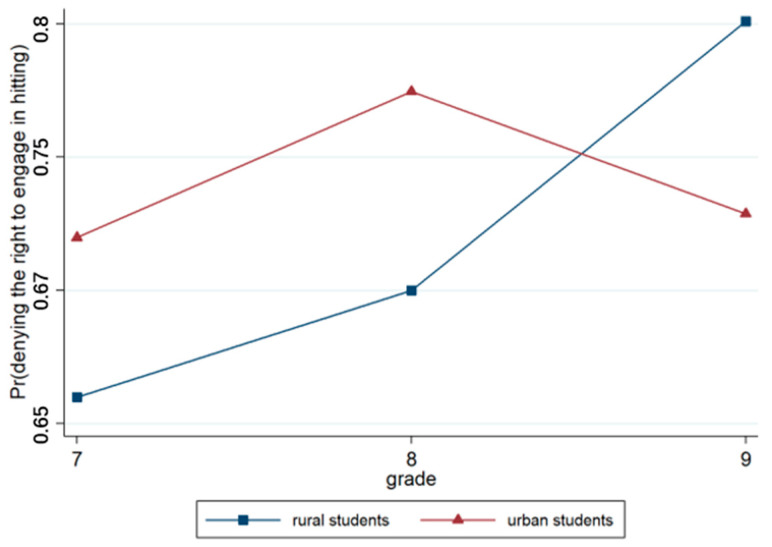
Interaction effect of region × grade.

Model 3 incorporated region and grade variables based on Model 2. The result showed that the effect of region was not significant, whereas the effect of grade was significant (*p* < 0.001). This finding indicated that senior students were more likely to deny the right to engage in hitting than junior students. Model 4 included the region × grade interaction terms, and the results revealed that the region × grade approach was highly significant (*p* < 0.001), as illustrated in Figure 6.

#### 4.2.2. Moral Judgments Concerning the Issue of Indirect Stealing

**Figure 7:**
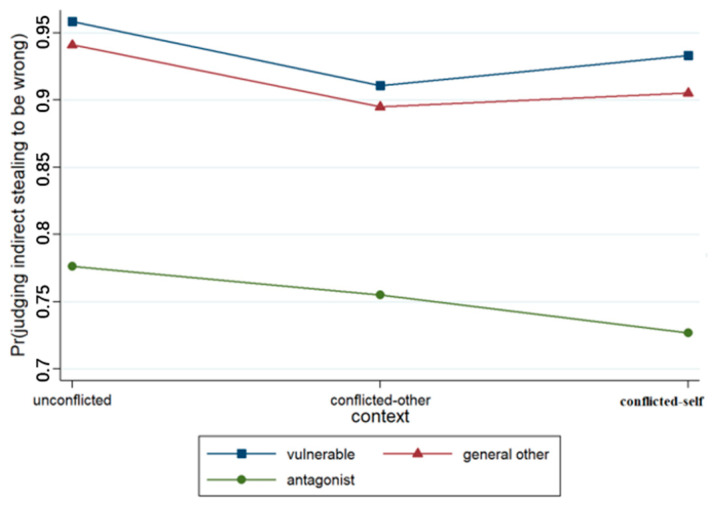
Interaction effect of context × relationship.

An ordered multiple logistic regression model was established on the basis of the scores pertaining to each question about indirect stealing. The variables and their interaction terms were successively incorporated into the model, and four models were obtained (see Table 3). Model 1 included the control variable of gender and two situational variables. The output results revealed that context (*p* < 0.001) and relationships (*p* < 0.001) had significant effects on the judgments made by junior high school students, as illustrated in Figure 7. The results concerning Model 2 indicated that the effect of context × relationship was not significant.

**Figure 8:**
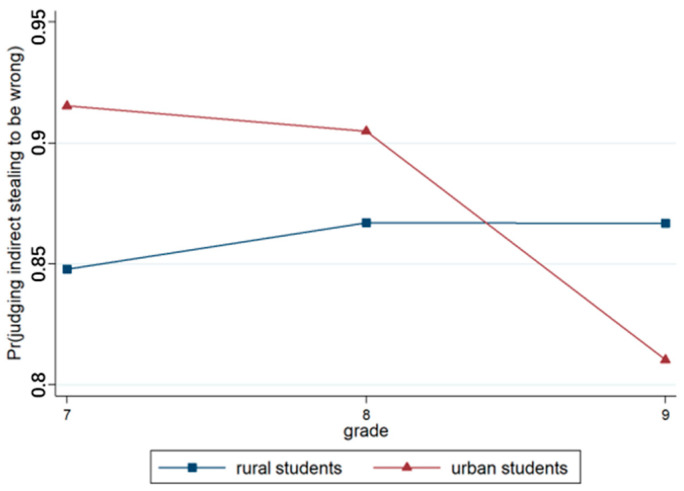
Interaction effect of region × grade.

Model 3 incorporated the region and grade variables based on Model 2. The output revealed that the effects of region and grade were significant (*p* < 0.001). Urban students were more likely to judge the action of “not returning money” to be wrong than rural students. Additionally, older students were more likely to judge this action to be acceptable than younger students. The results concerning Model 4 revealed that the region × grade interaction term was highly significant (*p* < 0.001), as illustrated in Figure 8.

**Figure 9:**
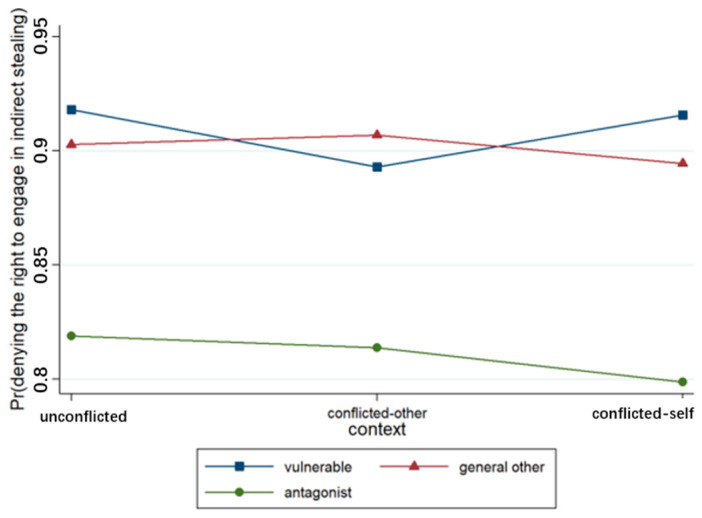
Interaction effect of context × relationship.

A binary logistic regression model was developed concerning participants’ judgments regarding the right to indirect stealing (see Table 4). The output results concerning Model 1 indicated that the effect of context was not significant, whereas the effect of relationship was significant (*p* < 0.001). The likelihood of participants affirming the right to keep the money increased when the recipient of the act changed from a vulnerable other to an antagonist, as illustrated in Figure 9. Model 2 included the context × relationship interaction term, and the results revealed that the context × relationship term was not significant.

**Figure 10:**
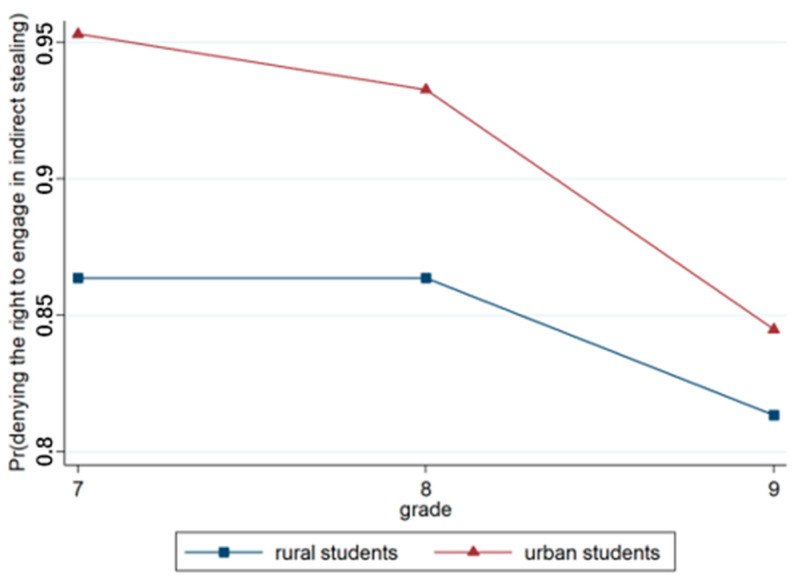
Interaction effect of region × grade.

Model 3, which was based on Model 2, incorporated region and grade variables. The output results revealed that the effect of region was significant (*p* < 0.001), with urban students more likely to deny the right to keep money than rural students. The effect of grade was significant, with older students more likely to assert the right to keep their money than younger students. Model 4 included the region × grade interaction terms, and the results revealed that the region × grade approach was highly significant (*p* < 0.001), as illustrated in Figure 10.

#### 4.2.3. Moral Judgments Concerning the Issue of Denying Help

**Figure 11:**
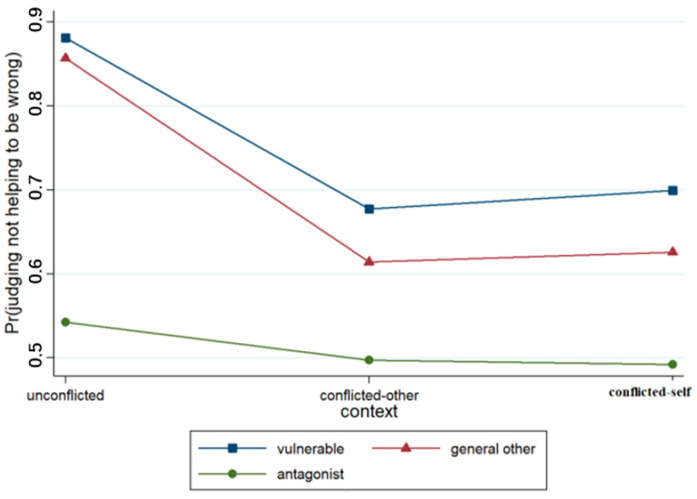
Interaction effect of context × relationship.

The variables and their interaction terms were successively incorporated into the model, and four models were obtained (see Table 5). In Model 1, only the control variable of gender and two situational variables were included. The results revealed that context (*p* < 0.001) and relationship (*p* < 0.001) had significant effects. The likelihood that junior high school students would deny the help decreased with the emergence of conflict as well as a change in the recipient of the act from a vulnerable other to an antagonist. Model 2 included the interaction term context × relationship, and the results revealed that the context × relationship term was highly significant (*p* < 0.001). As shown in Figure 11, there was little difference between junior high school students’ judgments of not helping the vulnerable and not helping the general other. Nearly half of the students judged helping the antagonist as acceptable, and the difference between the judgment of not helping the antagonist and the judgment of not helping others (the vulnerable and the general other) decreased with the emergence of conflict.

**Figure 12:**
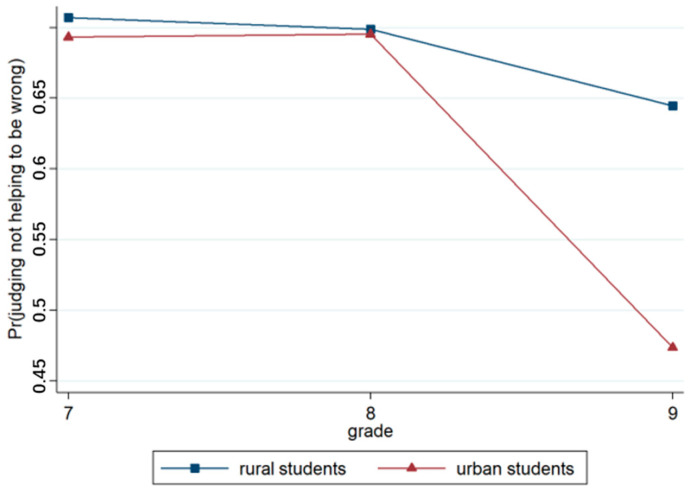
Interaction effect of region × grade.

Model 3, which was based on Model 2, incorporated region and grade variables. The output results concerning this model indicated that the effects of region (*p* < 0.001) and grade (*p* < 0.001) were significant. Urban students were more likely to judge the act of not helping as acceptable than rural students. Older students were more likely to judge the act of not helping as acceptable than younger students. The results concerning Model 4 revealed that the region × grade interaction term was significant (*p* < 0.001), as illustrated in Figure 12.

**Figure 13:**
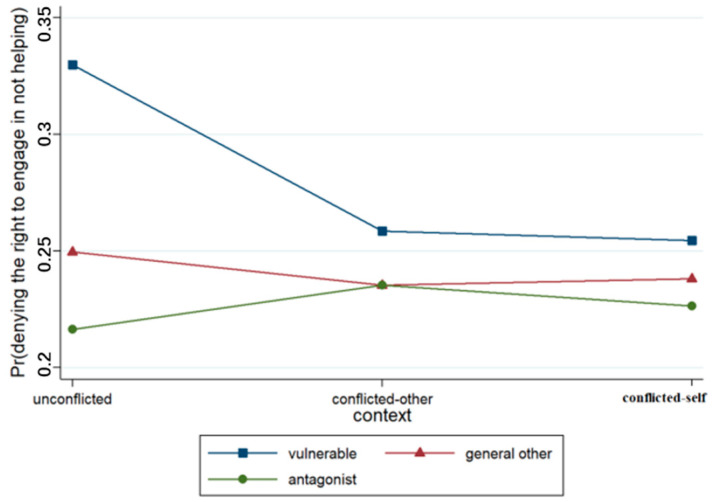
Interaction effect of context × relationship.

A binary logistic regression model was developed on the basis of participants’ judgments regarding the right to not help (see Table 6). The output results concerning Model 1 revealed that the effect of context was not significant, while the effect of the relationship was significant (*p* < 0.001). The likelihood that junior high school students would deny the right to not help decreased when the recipient of the act changed from a vulnerable other to a general other or an antagonist. Model 2 included the context × relationship interaction terms, and the results revealed that the context × relationship term was significant (*p* < 0.05), as illustrated in Figure 13.

**Figure 14:**
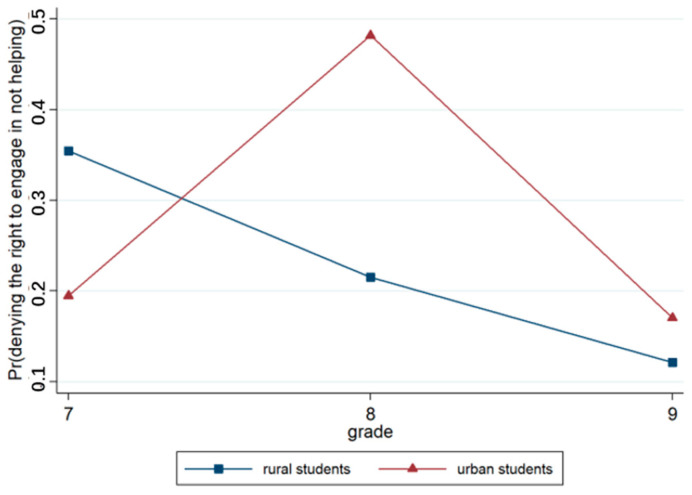
Interaction effect of region × grade.

Model 3, which was based on Model 2, incorporated region and grade variables. The output results regarding this model revealed that the effects of region (*p* < 0.001) and grade (*p* < 0.001) were significant. Urban students were more likely to judge that one does not have the right to not help than rural students. The grade effect was significant, and senior students tended to have a more positive attitude towards the right of behaviors that do not help those in need compared to lower-grade students. The results concerning Model 4 revealed that the region × grade approach was highly significant (*p* < 0.001). As shown in Figure 14, there was almost no difference in judgments between students in grades 7 and 8. Grade 9 students were less likely to judge not helping to be wrong than students in grades 7 and 8. Moreover, there was a large difference in judgments between urban and rural students, and more than half of the urban students in grade 9 judged the acts of not helping as acceptable.

## 5. Discussion

### 5.1. The Universality of Moral Judgments

The present study provides empirical evidence to support the domain theory, which posits that most junior high school students can make moral judgments in situations that do not involve conflict. Situational factors play a crucial role in shaping children’s moral judgments. Specifically, the presence of an antagonist recipient significantly increases students’ tendency to endorse the right to engage in the act in question. This finding is consistent with the results of previous research suggesting that individual discretion in decision-making is expanded and refined during early adolescence (Smetana, 2006).

Recent studies on the act of hurting others (Wang et al., 2010; Jambon & Smetana, 2014) have confirmed that most Chinese teenagers can make moral judgments in unambiguous situations. However, Chinese students are more likely to accept the acts of striking back against an aggressor and failing to return dropped money if the person who dropped the money is perceived as an antagonist. These results are in line with the conclusions of previous research on the effects of victim characteristics on children’s acceptance of non-moral behavior (Smetana & Ball, 2017). In most situations, Chinese students believe that failing to help others who are in need is morally wrong, which is consistent with findings reported by studies that have investigated American students (Nucci et al., 2018).

With respect to the consistency of teenagers’ judgments across different situations, Chinese junior high school students’ preferences for the personal domain increase as they enter grade 8, with students in grade 9 exhibiting a stronger tendency to protect the personal domain than students in grades 7 and 8. Few differences are observed between students in grades 7 and 8 in terms of their judgments concerning unethical actions and the right to engage in those actions under identical circumstances. However, in situations involving conflicts, grade-level differences are observed in students’ moral judgments, thus indicating that significant developmental changes occur in the moral judgments of teenagers across different stages (Chiasson et al., 2017; Smetana & Ball, 2019). The transitional period of moral development occurs at approximately the age of 14, at which point students begin to exhibit stronger preferences for the personal domain when conflicts occur between the personal and moral domains.

### 5.2. The Cultural Specificity of Moral Judgments

The findings of this study are in line with the results of previous research on the cultural adaptation of the domain theory, thus demonstrating that the content of the moral and personal domains varies across different cultural contexts and that the moral reasoning exhibited by Chinese students is influenced by their cultural environment.

With respect to acts of aggression, the contextual effects observed in this study differ from those reported in a previous study conducted in the United States, which indicated no significant differences in children’s judgments between self-conflicted and other conflicted situations. In contrast, the present study reveals a significant difference between the judgments made by Chinese students in these two types of situations. A larger proportion of Chinese students believe that one has the right to retaliate in situations involving self-conflict. Specifically, with regard to the question of whether individuals have the right to hit back when they are attacked by a general other or an antagonist, nearly half of the junior high school students included in this research claimed that individuals have such a right; furthermore, this view was more prevalent among younger students than among older students. This finding suggests that Chinese students possess a strong sense of self-defense; although they believe that hitting a general other unprovoked is wrong, they accept that doing so when one has been attacked is justifiable to some extent, thus highlighting the substantial conflict between the personal and moral domains.

In situations involving indirect theft, nearly all the students included in this research agreed that regardless of whether one or one’s friend needs money, one has no right to keep money dropped by a general other or a vulnerable person. Interestingly, a significant curvilinear relationship with age was observed, with a higher percentage of 14-year-old participants (49%) than younger participants (8-year-olds 20%; 11-year-olds 23%) or older participants (16-year-olds 15%) maintaining that the protagonist has the right to keep the money, as it no longer belongs to the person who dropped it. American scholars have distinguished this event from acts of aggression, as many students at approximately the age of 14 perceive keeping such money as an autonomous choice that involves greater conflicts between personal and moral goals, thus contributing to the expansion of the personal domain. However, the current study reveals that Chinese junior high school students across all grades and regions accurately judge that an individual has no right to keep money that has been dropped by another, thus indicating that the moral implications of this issue are stronger than those associated with hitting.

In judgments concerning whether to help someone in distress, Chinese junior middle school students’ views on the right to not help appear to be more consistent than those of American 14-year-olds, and the views vary only minimally with changes in situational characteristics. Conversely, the judgments made by American teenagers are more flexible with respect to situational factors. Most Chinese students believe that one has the right to not help. In most situations, the majority of these students judge the act of not helping to be wrong. However, the belief that the action is wrong is higher among American junior high school students than among Chinese students. Moreover, in the American study, the percentage of 14-year-olds who judged the action in question to be wrong was notably lower than the corresponding percentages among 11- and 16-year-olds. Chinese junior high school students view helping as more closely related to the personal domain, and when such students face conflicts between the personal and moral domains, they are more likely to prioritize the personal domain than American students. This pattern of judgment is associated with the prosocial attribute of helping, as most Chinese people perceive helping to represent an act of kindness. However, frequent reports of extortion schemes in Chinese society cause many individuals to avoid engaging in such acts of kindness.

The findings also highlight the prosocial aspect of helping, thus revealing a significant expansion of the personal domain among junior high school students. Due to the conflicts between these two domains, individuals’ moral judgments vary across different situations, thus highlighting middle school students’ ability to balance situational factors. The findings of some studies may partially explain this phenomenon. Previous research has proposed that numerous motives that have diverse emotional implications influence the altruistic behavior exhibited by children or adolescents in the context of decision-making tasks and that the relative significance of these motives shifts throughout the process of development (Krettenauer et al., 2019). Children’s assessments of helping situations evolve systematically on the basis of the recipient’s need for assistance and the potential costs faced by the helper. In cases in which a peer could be helped at minimal cost, children experience a moral obligation to help, independent of peer norms, parental authority, and reciprocity considerations. However, when the need for help and the associated costs are high, adolescents exhibit certain trade-offs in terms of their judgments (Sierksma et al., 2014). As they mature, young people increasingly begin to focus on the consequences of providing help (Recchia et al., 2015).

### 5.3. Differences in Moral Judgment Between Students from Urban and Rural Areas

The findings suggest that grades 8–9 serve as a transitional phase in the moral development of Chinese students from both urban and rural areas. When such students encounter conflicts between the personal and moral domains, urban students are more likely to protect the personal domain. The inclination of urban students to defend the personal domain is evidenced by the fact that higher percentages of rural students in grades 7 and 8 prioritize the personal domain. Students in grades 7 and 8 exhibit less pronounced preferences for the personal domain in both urban and rural areas than students in grade 9.

Moreover, a marked disparity is evident between urban and rural students in grade 9 in terms of their awareness of autonomy. Although urban students in grade 9 make a similar percentage of judgments concerning the rightness of a given action, these students also exhibit a robust awareness of the importance of protecting personal rights and a tendency to support such actions. In conflicts between the personal and moral domains, the tendency to prioritize the personal domain is notable. Moreover, urban students in grade 8 tend to deny the right to perform the acts in question. However, their inclination to view this act as wrong does not differ significantly from that of urban students in grade 7 or rural students in grade 8. This finding suggests that urban students in grade 8 exhibit a stronger sense of prioritizing individual rights than their rural counterparts.

The urban and rural differences observed in junior high school students’ moral judgments, as revealed by the findings of this research, corroborate the results of previous studies that have highlighted variations in moral culture within a given society (Graham et al., 2016; Zhao & Selman, 2020). These findings imply that moral development is shaped by local socialization processes. Chinese teenagers’ reasoning concerning individual rights is influenced by their environmental background, in which context urban teenagers are more inclined to protect individual autonomy (Lahat et al., 2009; Liu & Yang, 2017). Previous research has offered explanations for these findings, indicating that urban teenagers exhibit a strong sense of self and are prone to defend their own rights and interests (Jiang, 2006; Yang & Wang, 2018). To a certain extent, Liu et al. explained the divergent performance exhibited by rural children in terms of their judgments concerning their personal rights and the rightness or wrongness of a given action. Rural left-behind children struggle to determine the scope of individual responsibility and individual autonomy and are more likely to deny rules in the personal domain; furthermore, as male students age, the range of their personal domain is reduced, the autonomy of their actions decreases, and they are therefore more likely to make judgments in the moral domain based on rules (Liu & Yang, 2017). In contrast, the findings of previous research (Chi, 2005; Zhu, 2007; Du, 2012) regarding the moral issues that characterize rural left-behind children (e.g., a tendency to lack moral cognition, a weak moral character, abnormal moral behavior, moral decline, character flaws, and distorted values) are not consistently reflected in this study.

## 6. Conclusions

This study demonstrates the universality and cultural specificity of moral judgments made by Chinese junior high school students. The results confirm the domain theory and its applicability in different cultural contexts. However, it is essential to recognize the fact that the content and scope of moral and personal domains may vary across cultures. Moral judgments made by Chinese students are influenced by their cultural environment and the specific situations that they encounter. This knowledge may be valuable for educators and policy-makers seeking to design interventions and educational programs aimed at promoting moral development and fostering ethical behavior in adolescents.

This study has several limitations. First, the sample that this research focused on included students from only three grades in junior middle school, and cross-sectional data were used in this study. It was thus impossible to perform systematic comparative research on students’ moral judgments in terms of developmental trends. In the future, researchers can conduct a comprehensive investigation of children’s moral judgment across broader age ranges. Second, this study failed to obtain a random sample, and the conclusions of this research must be verified by reference to a larger sample and by employing more reasonable methods. In addition, this study took the form of a closed questionnaire survey, and the results of this research thus do not reflect the reasons underlying moral judgments made by and the reasoning process employed by junior high school students. In the future, in-depth research can be conducted to enrich our understanding of these research questions.

## Figures and Tables

**Table 1:** Ordered multiple logistic models for judgments concerning the rightness or wrongness of hitting.

	Model (1)	Model (2)	Model (3)	Model (4)
Gender	−0.142 **	−0.142 *	−0.152 **	−0.173 **
	(0.055)	(0.055)	(0.056)	(0.056)
Context	−0.802 ***	−1.180 ***	−1.179 ***	−1.194 ***
	(0.035)	(0.101)	(0.101)	(0.101)
Relationship	−0.792 ***	−1.168 ***	−1.168 ***	−1.182 ***
	(0.035)	(0.101)	(0.101)	(0.101)
Context × Relationship		0.175 ***	0.175 ***	0.177 ***
		(0.044)	(0.044)	(0.044)
Region			−0.017	4.080 ***
			(0.056)	(0.552)
Grade			0.059	0.285 ***
			(0.034)	(0.046)
Region × Grade				−0.514 ***
				(0.069)
N	5211	5211	5211	5211
Pseudo R2	0.076	0.077	0.077	0.081

Note: *** *p* < 0.001, ** *p* < 0.01, * *p* < 0.05; the figures presented in brackets are standard errors.

**Table 2:** Ordered multiple logistic models for judgments concerning the right to engage in hitting.

	Model (1)	Model (2)	Model (3)	Model (4)
Gender	0.211 **	0.210 **	0.238 ***	0.255 ***
	(0.068)	(0.068)	(0.069)	(0.069)
Context	1.046 ***	1.653 ***	1.663 ***	1.669 ***
	(0.045)	(0.140)	(0.141)	(0.141)
Relationship	0.560 ***	1.197 ***	1.203 ***	1.207 ***
	(0.043)	(0.145)	(0.145)	(0.145)
Context × Relationship		−0.273 ***	−0.274 ***	−0.274 ***
		(0.058)	(0.059)	(0.059)
Region			−0.122	−3.259 ***
			(0.069)	(0.678)
Grade			−0.245 ***	−0.418 ***
			(0.042)	(0.057)
Region × Grade				0.394 ***
				(0.085)
N	5184	5184	5184	5184
Pseudo R2	0.130	0.134	0.140	0.143

Note: *** *p* < 0.001, ** *p* < 0.01; the figures presented in brackets are standard errors.

**Table 3:** Ordered multiple logistic model for judgments concerning the rightness or wrongness of indirect stealing.

	Model (1)	Model (2)	Model (3)	Model (4)
Gender	−0.252 ***	−0.252 ***	−0.211 **	−0.232 ***
	(0.069)	(0.069)	(0.069)	(0.069)
Context	−0.171 ***	−0.415 **	−0.415 **	−0.417 **
	(0.042)	(0.136)	(0.137)	(0.137)
Relationship	−0.874 ***	−1.091 ***	−1.099 ***	−1.106 ***
	(0.045)	(0.125)	(0.125)	(0.125)
Context × Relationship		0.104	0.104	0.105
		(0.055)	(0.056)	(0.056)
Region			0.309 ***	3.884 ***
			(0.070)	(0.706)
Grade			−0.177 ***	−0.004
			(0.042)	(0.054)
Region × Grade				−0.443 ***
				(0.087)
N	5153	5153	5153	5153
Pseudo R2	0.051	0.052	0.056	0.059

Note: *** *p* < 0.001, ** *p* < 0.01; the figures presented in brackets are standard errors.

**Table 4:** Ordered multiple logistic models for judgments concerning the right to engage in indirect stealing.

	Model (1)	Model (2)	Model (3)	Model (4)
Gender	0.553 ***	0.553 ***	0.483 ***	0.502 ***
	(0.088)	(0.089)	(0.090)	(0.090)
Context	0.048	−0.011	−0.014	−0.015
	(0.052)	(0.155)	(0.156)	(0.156)
Relationship	0.462 ***	0.408 **	0.414 **	0.414 **
	(0.054)	(0.143)	(0.144)	(0.144)
Context × Relationship		0.027	0.028	0.029
		(0.066)	(0.066)	(0.066)
Region			−0.612 ***	−4.911 ***
			(0.091)	(1.000)
Grade			0.367 ***	0.202 **
			(0.054)	(0.065)
Region × Grade				0.521 ***
				(0.120)
N	5142	5142	5142	5142
Pseudo R2	0.030	0.030	0.054	0.059

Note: *** *p* < 0.001, ** *p* < 0.01; the figures presented in brackets are standard errors.

**Table 5:** Ordered multiple logistic models for judgments concerning the rightness or wrongness of denying help.

	Model (1)	Model (2)	Model (3)	Model (4)
Gender	0.553 ***	0.553 ***	0.483 ***	0.502 ***
	(0.088)	(0.089)	(0.090)	(0.090)
Context	0.048	−0.011	−0.014	−0.015
	(0.052)	(0.155)	(0.156)	(0.156)
Relationship	0.462 ***	0.408 **	0.414 **	0.414 **
	(0.054)	(0.143)	(0.144)	(0.144)
Context × Relationship		0.027	0.028	0.029
		(0.066)	(0.066)	(0.066)
Region			−0.612 ***	−4.911 ***
			(0.091)	(1.000)
Grade			0.367 ***	0.202 **
			(0.054)	(0.065)
Region × Grade				0.521 ***
				(0.120)
N	5142	5142	5142	5142
Pseudo R2	0.030	0.030	0.054	0.059

Note: *** *p* < 0.001, ** *p* < 0.01; the figures presented in brackets are standard errors.

**Table 6:** Ordered multiple logistic models for judgments concerning the right to not help.

	Model (1)	Model (2)	Model (3)	Model (4)
Gender	0.071	0.071	0.007	−0.024
	(0.065)	(0.065)	(0.066)	(0.067)
Context	0.069	0.284 **	0.290 **	0.293 **
	(0.040)	(0.104)	(0.105)	(0.105)
Relationship	0.147 ***	0.365 ***	0.372 ***	0.376 ***
	(0.040)	(0.105)	(0.106)	(0.107)
Context × Relationship		−0.110 *	−0.112 *	−0.113 *
		(0.049)	(0.049)	(0.050)
Region			−0.230 ***	4.644 ***
			(0.066)	(0.651)
Grade			0.388 ***	0.695 ***
			(0.041)	(0.059)
Region × Grade				−0.619 ***
				(0.082)
N	5062	5062	5062	5062
Pseudo R2	0.003	0.004	0.022	0.033

Note: *** *p* < 0.001, ** *p* < 0.01, * *p* < 0.05; the figures presented in brackets are standard errors.

## Data Availability

Data is contained within the article.

## Appendix A

**Table A1:** Percentage of participants who judged hitting, indirect stealing, and not helping to be wrong by context and relationship across different grades and regions.

Context/Relationship		Hitting				Indirect Stealing				Not Helping			
		Grade 7	Grade 8	Grade 9	Total	Grade 7	Grade 8	Grade 9	Total	Grade 7	Grade 8	Grade 9	Total
Unconflicted													
Vulnerable	Rural	94.17	98.21	95.54	95.91	95.65	98.21	99.12	97.65	90.43	93.64	89.29	91.10
	Urban	97.65	94.25	93.26	95.02	97.65	94.12	89.89	93.82	95.24	85.71	74.16	84.80
General other	Rural	96.58	95.54	99.12	97.08	93.10	98.21	94.69	95.31	90.40	89.19	89.38	89.68
	Urban	97.65	95.45	89.89	94.27	98.82	94.25	86.36	93.08	89.41	83.12	68.54	80.08
Antagonist	Rural	59.32	58.93	68.14	62.10	72.41	74.11	76.99	74.49	55.65	58.56	55.75	56.64
	Urban	64.29	79.31	65.17	69.62	85.88	89.53	71.91	82.31	49.41	64.94	43.82	52.19
Self-conflicted													
Vulnerable	Rural	75.21	71.43	86.73	77.78	92.17	96.36	92.92	93.79	78.26	72.73	68.14	73.08
	Urban	78.57	73.86	59.09	70.38	96.47	91.46	88.76	92.19	82.35	75.32	46.07	67.33
General other	Rural	51.72	40.18	62.83	51.61	92.98	90.99	87.50	90.50	72.17	68.81	56.64	65.88
	Urban	50.00	55.68	48.31	51.34	95.29	91.57	83.15	89.88	68.24	66.23	47.19	60.16
Antagonist	Rural	35.04	30.36	51.33	38.89	72.17	63.06	70.80	68.73	53.91	48.18	44.25	48.82
	Urban	44.71	51.14	41.57	45.08	78.82	85.37	69.66	77.73	51.76	63.64	39.33	51.00
Other conflicted													
Vulnerable	Rural	75.21	70.54	83.19	76.32	88.70	92.86	89.38	90.29	77.39	72.97	71.43	73.96
	Urban	80.00	78.41	60.67	72.90	97.65	91.36	86.52	91.76	70.24	68.83	41.57	59.60
General other	Rural	49.14	47.32	65.49	53.96	91.30	89.29	88.50	89.71	70.43	71.17	57.14	66.27
	Urban	55.29	65.91	51.69	57.63	92.94	91.36	83.15	89.02	69.41	61.04	38.20	55.78
Antagonist	Rural	42.74	33.04	53.15	42.94	72.17	69.64	72.57	71.47	54.78	51.35	47.32	51.18
	Urban	50.59	57.95	41.57	50.00	83.53	87.65	73.03	81.18	54.12	62.34	33.71	49.40

**Table A2:** Percentage of participants who judged that one would not have the right to engage in hitting, indirect stealing, and not helping by context and relationship across different grades and regions.

Context/Relationship		Hitting				Indirect Stealing				Not Helping			
		Grade 7	Grade 8	Grade 9	Total	Grade 7	Grade 8	Grade 9	Total	Grade 7	Grade 8	Grade 9	Total
Unconflicted													
Vulnerable	Rural	95.69	98.21	98.23	97.36	92.98	92.86	88.50	91.45	45.61	31.53	16.07	31.16
	Urban	96.47	96.47	95.45	96.12	97.62	95.35	84.27	92.28	28.24	54.55	21.35	33.86
General other	Rural	98.28	99.10	96.46	97.94	93.04	89.29	81.42	87.94	35.96	19.82	9.73	21.89
	Urban	98.82	95.45	91.01	95.04	98.82	95.35	84.27	92.69	20.00	50.65	16.85	28.29
Antagonist	Rural	66.96	72.32	86.73	75.29	77.39	75.00	78.76	77.06	23.68	18.92	12.39	18.34
	Urban	75.29	85.06	86.52	82.38	90.48	90.59	83.15	87.98	9.52	49.35	19.10	25.20
Self-conflicted													
Vulnerable	Rural	71.93	72.32	81.42	75.22	92.11	92.73	86.84	90.21	36.28	20.91	11.61	22.99
	Urban	70.24	75.00	60.67	68.58	97.59	96.39	87.64	93.73	22.35	47.37	16.85	28.00
General other	Rural	34.51	25.89	45.05	35.12	93.04	88.29	79.46	86.98	36.84	22.73	7.96	22.55
	Urban	33.33	54.55	50.56	46.36	98.81	95.12	83.15	92.16	17.65	44.74	14.61	24.80
Antagonist	Rural	36.28	43.75	65.49	48.52	76.99	71.17	70.80	73.00	28.95	19.09	11.61	19.94
	Urban	54.12	61.36	51.69	55.73	91.76	92.77	82.02	88.72	17.86	46.05	16.85	26.10
Other conflicted													
Vulnerable	Rural	83.48	85.71	88.50	85.88	91.23	89.29	80.53	87.02	40.71	20.72	14.29	25.30
	Urban	80.00	85.23	79.78	81.68	92.94	91.36	88.76	90.98	20.00	48.00	14.61	26.51
General other	Rural	57.02	65.18	81.25	67.75	91.23	88.39	84.96	88.20	36.04	18.02	8.04	20.66
	Urban	68.24	79.55	74.16	74.05	97.65	93.83	87.64	92.94	21.18	46.05	6.61	26.40
Antagonist	Rural	51.75	58.93	77.68	62.72	77.88	74.11	75.22	75.74	33.63	18.92	14.29	22.32
	Urban	68.24	72.09	69.32	69.88	92.94	91.25	82.02	88.58	15.29	45.33	16.85	24.90

## References

[B1-behavsci-15-00187] Chi X. (2005). 留守儿童道德成长问题的心理社会分析 [Psychosocial analysis of the moral growth of left-behind children]. Research on Teacher Education.

[B2-behavsci-15-00187] Chiasson V., Vera-Estay E., Lalonde G., Dooley J. J., Beauchamp M. H. (2017). Assessing social cognition: Age-related changes in moral reasoning in childhood and adolescence. The Clinical Neuropsychologist.

[B3-behavsci-15-00187] Dahl A., Gingo M., Uttich K., Turiel E. (2018). Moral reasoning about human welfare in adolescents and adults: Judging conflicts involving sacrificing and saving lives: I. Introduction. Monographs of the Society for Research in Child Development.

[B4-behavsci-15-00187] Du H. (2012). 农村留守儿童道德成长偏差之思考—以佛山地区为例 [Reflections on the moral growth deviation of left-behind children in rural areas—A case study of foshan]. Academic Exploration.

[B5-behavsci-15-00187] Fang G., Fang F. X., Keller M., Edelstein W., Kehle T. J., Bray M. A. (2003). Social moral reasoning in chinese children: A developmental study. Psychology in the Schools.

[B6-behavsci-15-00187] Graham J., Meindl P., Beall E., Johnson K. M., Zhang L. (2016). Cultural differences in moral judgment and behavior, across and within societies. Current Opinion in Psychology.

[B7-behavsci-15-00187] Hu X. (2018). Global orientations and moral foundations: A cross-cultural examination among American, Chinese, and international students.

[B8-behavsci-15-00187] Hu X., Zhu Y., Yu F., Wilder D. A., Zhang L., Chen S. X., Peng K. (2020). A cross-cultural examination on global orientations and moral foundations. PsyCh Journal.

[B9-behavsci-15-00187] Jambon M., Smetana J. G. (2014). Moral complexity in middle childhood: Children’s evaluations of necessary harm. Developmental Psychology.

[B10-behavsci-15-00187] Jiang Y. (2006). 未成年人群体与成年人群体道德状况比较 [Comparison of moral status between minors and adults]. Contemporary Youth Research.

[B11-behavsci-15-00187] Killen M., McGlothlin H., Lee-Kim J., Keller H., Poortinga Y. H., Schölmerich A. (2002). Between individuals and culture: Individuals’ evaluations of exclusion from social groups. Between culture and biology: Perspectives on ontogenetic development.

[B12-behavsci-15-00187] Krettenauer T., Bauer K., Sengsavang S. (2019). Fairness, prosociality, hypocrisy, and happiness: Children’s and adolescents’ motives for showing unselfish behaviour and positive emotions. British Journal of Developmental Psychology.

[B13-behavsci-15-00187] Lahat A., Helwig C. C., Yang S., Tan D., Liu C. (2009). Mainland Chinese adolescents’ judgments and reasoning about self-determination and nurturance rights. Social Development.

[B14-behavsci-15-00187] Liu J., Yang S. (2017). 留守儿童的道德判断及其对道德教育的启示—基于社会认知领域理论的视角 [Moral judgment of left-behind children and its enlightenment to moral education—From the perspective of social cognition domain theory]. Open Times.

[B15-behavsci-15-00187] López-Pérez B., Gummerum M., Keller M., Filippova E., Gordillo M. V. (2015). Sociomoral reasoning in children and adolescents from two collectivistic cultures. European Journal of Developmental Psychology.

[B16-behavsci-15-00187] Nucci L. (2001). Education in the moral domain.

[B17-behavsci-15-00187] Nucci L., Turiel E. (2009). Capturing the complexity of moral development and education. Mind, Brain, and Education.

[B18-behavsci-15-00187] Nucci L., Turiel E., Roded A. D. (2018). Continuities and discontinuities in the development of moral judgments. Human Development.

[B19-behavsci-15-00187] Recchia H. E., Wainryb C., Bourne S., Pasupathi M. (2015). Children’s and adolescents’ accounts of helping and hurting others: Lessons about the development of moral agency. Child Development.

[B20-behavsci-15-00187] Sachdeva S., Singh P., Medin D. (2011). Culture and the quest for universal principles in moral reasoning. International Journal of Psychology.

[B21-behavsci-15-00187] Sierksma J., Thijs J., Verkuyten M., Komter A. (2014). Children’s reasoning about the refusal to help: The role of need, costs, and social perspective taking. Child Development.

[B22-behavsci-15-00187] Smetana J. G., Kail R. V., Reese H. W. (2002). Culture, autonomy, and personal jurisdiction in adolescent-parent relationships. Advances in child development and behavior.

[B23-behavsci-15-00187] Smetana J. G., Killen M., Smetana J. G. (2006). Social-cognitive domain theory: Consistencies and variations in children’s moral and social judgments. Handbook of moral development.

[B24-behavsci-15-00187] Smetana J. G., Ball C. L. (2017). Young children’s moral judgments, justifications, and emotion attributions in peer relationship contexts. Child Development.

[B25-behavsci-15-00187] Smetana J. G., Ball C. L. (2019). Heterogeneity in children’s developing moral judgments about different types of harm. Developmental Psychology.

[B26-behavsci-15-00187] Sommer M., Meinhardt J., Rothmayr C., Döhnel K., Hajak G., Rupprecht R., Sodian B. (2014). Me or you? Neural correlates of moral reasoning in everyday conflict situations in adolescents and adults. Society for Neuroscience.

[B27-behavsci-15-00187] Thoma S. J., Walker D. I., Chen Y.-H., Frichand A., Moulin-Stożek D., Kristjánsson K. (2019). Adolescents’ application of the virtues across five cultural contexts. Developmental Psychology.

[B28-behavsci-15-00187] Turiel E. (2002). The culture of morality: Social development, context, and conflict.

[B29-behavsci-15-00187] Turiel E. (2008). The Development of children’s orientations toward moral, social, and personal orders more than a sequence in development. Human Development.

[B30-behavsci-15-00187] Wang M. F., Yan X. M., Yao L. (2010). Children and adolescents’ moral judgments about physical and relational aggression. Chinese Journal of Clinical Psychology.

[B31-behavsci-15-00187] Yang S., Wang Q. (2018). 中学生价值观念的实证研究与思考 [Empirical research and reflection on values of middle school students]. Moral Education in Primary and Secondary Schools.

[B32-behavsci-15-00187] Zhang Y., Li S. (2015). Two measures for cross-cultural research on morality: Comparison and revision. Psychological Reports.

[B33-behavsci-15-00187] Zhao X., Selman R. L. (2020). Bystanders’ responsibilities in a situation of teasing: A dual dynamic analysis (DDA) for Understanding culture, context, and youth moral development. Qualitative Psychology.

[B34-behavsci-15-00187] Zhu H. (2007). 关于当前农村留守儿童德育的思考 [Reflections on the moral education of current left-behind children in rural areas]. Modern Education Science.

